# A retrospective metagenomic analysis of fecal microbiota transplantation donors from five countries: Safety considerations for donor screening and core microbiome profiles of qualified donors

**DOI:** 10.71150/jm.2604010

**Published:** 2026-09-30

**Authors:** Sha-Sha Li, Yun-Hui Niu, Hong-Jing Yu

**Affiliations:** Shanghai Engineering Research Center of Innovative Probiotic Drugs, SPH Sine Pharmaceutical Co., Ltd., Shanghai 201206, P. R. China

**Keywords:** fecal microbiota transplantation, donor screening, safety assessment, core microbiome

## Abstract

Fecal microbiota transplantation (FMT) has been successfully applied on clinical aspects, but its clinical outcomes remain unpredictable due to inconsistent donor screening protocols across hospitals, institutions, and countries. Hence, a retrospective analysis of metagenomic data from published studies on FMT donors via a unified bioinformatics workflow might contribute to the understanding of the safety considerations for donor screening and the fecal microbial profiles of qualified donors. In this study, we reanalyzed metagenomic data of 475 screened donor fecal samples from 24 studies spanning China, the USA, Canada, New Zealand, and the Netherlands. The genomic safety risks were evaluated by profiling antibiotic resistance genes (ARGs) and virulence factors (VFs), the results of which showed that no major toxin-associated virulence genes, such as Shiga toxin, Shiga-like toxin, or botulinum neurotoxin (BoNTs) genes harbored in the detected *Escherichia coli*, *Clostridium butyricum*, and *Streptococcus pneumoniae*, but several high-risk ARGs remained insufficiently addressed. The distribution of ARG-harboring bacteria in eligible FMT donors was country-specific. The alpha-diversity and microbial community structure were comparable between donor fecal samples from China and the USA. Interestingly, the core microbiome in fecal samples from Canada, the Netherlands, and New Zealand formed a single guild, while that from China and the USA formed two guilds, with predominantly positive intra-guild and negative inter-guild correlations, indicating that the co-abundance patterns of the core microbiome were conserved among certain countries. Furthermore, an exploratory retrospective classifier was developed based on core microbiome profiles to distinguish eligible FMT donors from general healthy individuals. These results provide evidence for integrating metagenomic sequencing into future FMT donor screening strategies.

## Introduction

FMT is used to modulate the recipient’s intestinal microbiome for therapeutic purposes by transferring gut microorganisms, and luminal content from qualified donors to recipients (Schmidt et al., 2022). It can be administered as a monotherapy to cure diseases or combined with other treatments to enhance therapeutic efficacy (Kao et al., 2021; Routy et al., 2023). To date, approximately 600 clinical studies registered on ClinicalTrials.gov have evaluated the efficacy or safety of FMT on a broad range of indications, such as recurrent *Clostridioides difficile* infection (rCDI), ulcerative colitis, melanoma, and acute graft-versus-host disease (Baruch et al., 2021; Baunwall et al., 2022; Paramsothy et al., 2017; Reddi et al., 2025). FDA has approved two live biotherapeutic products for rCDI treatment: REBYOTA (RBX2660) in December 2022 and VOWST (SER-109) in April 2023, underscoring the increasing clinical interests of FMT for treating diseases associated with gut microbiota dysregulation (Gonzales-Luna et al., 2023).

Indeed, recruitment and selection of stool donors for FMT is challenging (Papanicolas et al., 2020). These challenges are compounded by the intrinsic complexity and variability of stool as a biological material, which limits the safety and efficacy of FMT across different clinical contexts (Haifer et al., 2022; Papanicolas et al., 2020; Reddi et al., 2025; Yadegar et al., 2024). Meanwhile, beneficial commensal microorganisms (BCMs), which protect against pathogen colonization, facilitate colonic mucus development and its full protective function, and modulate bile acid metabolism, may contribute substantially to the efficacy of FMT (Holmberg et al., 2024; Liu et al., 2025a; Oliveira et al., 2020; Wortelboer et al., 2022).

Current FMT donor screening protocols rely on a combination of serological tests, stool bacteriological cultures, microscopic parasite examinations, and pathogen-specific PCR assays, most of which were modified from existing guidelines, rather than being tailored to microbiota transplantation (Lopetuso et al., 2023). While serological testing has achieved relative consistency, culture-based techniques remain labor-intensive, and molecular approaches face sensitivity limitations in clinical implementation (Goldsmith et al., 2024; Liu et al., 2025b). Although this organism-level strategy has substantially improved FMT safety, it does not fully capture all potential risks, owing to the potential horizontal transfer of ARGs (Lund et al., 2025; Maddamsetti et al., 2024). Some microbiota-based donor screening studies primarily focused on genus-level taxonomic characterization and alpha-diversity metrics, with limited implementation of strain-resolved genomic analyses or detection of high-risk ARGs for safety assessment (Zhang et al., 2023). Metagenomic sequencing represents a promising alternative for donor screening, as it enables simultaneous detection of both viable and non-viable pathogens, identification of ARGs, VFs, and comprehensive characterization of microbiota composition (Liu et al., 2025b). Wu et al. (2024) established a core-microbiome identification strategy based on de novo assembled high-quality metagenome-assembled genomes (HQMAGs), which was genome-specific, database-independent, and interaction-focused. This strategy exhibits robust performance in discriminating gut microbial communities between diseased and healthy states (Wu et al., 2024). Therefore, this strategy exhibits considerable potential in the application of microbiome characterization for FMT donors’ screening.

In this study, we conducted a comprehensive metagenomic analysis of gut microbiota of FMT donors from 24 independent studies. Firstly, we evaluated genomic safety risks by profiling ARGs and VFs. In parallel, we assessed cross-country variations of microbiome characteristics by analyzing alpha-diversity, taxonomic composition, DRMs, BCMs, and guilds formed by core microbiome. These results demonstrated that the distribution of ARG-harboring bacteria in eligible FMT donors was country-specific, whereas the co-abundance patterns of the core microbiome were conserved among the USA and China, as well as among New Zealand, Canada, and the Netherlands. Building on these descriptive findings, we further developed an exploratory retrospective classifier based on core microbiome profiles to distinguish eligible FMT donors from general healthy individuals. Collectively, this study presents a retrospective metagenomic survey describing the genomic safety profiles and community characteristics of FMT donor microbiomes, which might help inform future screening strategy development.

## Materials and Methods

### Metagenomic datasets of fecal microbiota transplantation donors

Gut metagenomic data of donors from 24 FMT public datasets were downloaded from SRA or ENA database. These datasets were selected as they met the following specific criteria (Table S1): i) openly published before October 2024 and accessible for download and reuse; ii) sufficiently detailed description on donor selection; iii) eligible adult donors were recruited following screening protocols that included, at a minimum, laboratory blood and fecal assessments. Finally, metagenomic data derived from 578 fecal samples of donors were identified.

### Metagenomic datasets of general healthy individuals

The public Chinese Microbiome Project (CMP) metagenomic dataset was downloaded from the NCBI SRA database (PRJNA801051), which including 239 fecal samples from healthy Chinese individuals (Zhang et al., 2022b). Following the exclusion of low-quality samples, 237 samples were retained for further analysis.

The fecal microbiome datasets of healthy individuals from the Human Microbiome Project (HMP) included in this research were downloaded from the NCBI Sequence Read Archive (SRA) database (accession: PRJNA48479) (Lloyd-Price et al., 2017). The SraRunTable.csv file was used to filtered out the non-gut metagenomic data. Samples were retained if (i) the isolation_source column was "G_DNA_Stool", (ii) the LibrarySource was "METAGENOMIC", (iii) the material type(exp) was neither "DNA Genomic / Genomic DNA" nor "DNA Somatic", and (iv) the bases were over 2 Gb. Finally, 287 samples were included for subsequent data analysis.

### Data quality control

Quality control of raw reads was performed using fastp (version 0.23.2) with default parameters, and subsequently, human sequences (GCF_000001405.40) were removed using KneadData (https://bitbucket.org/biobakery/kneaddata) (Chen et al., 2018). A total of 77 samples from donors were excluded from further analysis owing to that the ratio of high-quality base to raw-data base was less than 75% or the total amount of high-quality data was less than 2 Gb.

### Metagenome assembled genomes (MAGs) analysis

Metagenomic datasets from 403 donor samples with over 2,000 Gb quality-filtered data were used for MAGs analysis. The detailed analytical procedures included: i) De novo assembly of each sample was conducted by MEGAHIT (v1.1.2) (Li et al., 2015); ii) Contigs longer than 1,000 bp from the assembly results were subsequently processed for binning using Metabat (Version 2.12.1), Maxbin (Version 2.2.5), and CONCOCT (Version 0.5.0) (Kang et al., 2019; Wu et al., 2016); iii) The bins were dereplicated using dRep (Version 2.2.9) with parameters -pa 0.9 -sa 0.99 (Olm et al., 2017); iv) The quality of the bins was assessed using CheckM (Parks et al., 2015). Bins with contamination below 10% and completeness exceeding 90% were classified as high-quality MAGs (HQMAGs) and prioritized for subsequent analyses; v) The HQMAGs' abundance in each sample was determined using CoverM (Version 0.7.0) by metagenomic read recruitment to their genomes, followed by normalization of read counts using the RPKM (Reads Per Kilobase of genome per Million mapped reads) metric (Aroney et al., 2025). The taxonomic classification of the genomes was conducted using GTDB-Tk with default settings (Chaumeil et al., 2022).

### Taxonomic profiling

To characterize the microbial composition of donor feces, MetaPhlAn (v4.0.6) was employed for read-based taxonomic profiling of bacteria, archaea and eukaryote (Blanco-Míguez et al., 2023).

#### 1. DRMs list

DRMs refer to microbial species that are associated with various human diseases, either as pathogens or as contributors to disease progression. The DRMs List was generated by integrating the pathogenic microorganisms from the "List of Human Pathogenic Microorganisms" issued by the National Health Commission of the People's Republic of China (NHC), specifically those categorized as Risk Group I and II. Additionally, it incorporated the pathogens documented in the "Annual Epidemiological Report" of the European Centre for Disease Prevention and Control (ECDC), which can be accessed at https://www.ecdc.europa.eu/en/publications-data/monitoring/all-annual-epidemiological-reports, as well as the toxin-producing microorganisms related to the "Select Agents and Toxins" jointly maintained by the Department of Health and Human Services (HHS) and the United States Department of Agriculture (USDA), available at https://www.selectagents.gov/sat/list.htm. In this study, we investigated the abundance and distribution patterns of DRMs (Table S3) in the microbiome of FMT donors.

#### 2. BCMs list

The more commonly utilized BCMs have been approved for food applications by NHC as recorded in the List of Cultures Available in Food (last updated on January 2025); or accepted as having Generally Recognized as Safe status by the U.S. Food and Drug Administration, as documented on its official website (https://www.hfpappexternal.fda.gov/scripts/fdcc/index.cfm?set=GRASNotices; last updated 3 March 2025); or granted Qualified Presumption of Safety status by the European Food Safety Authority, as specified in the List of Microorganisms with QPS Status (updated until March 2024).

Besides, using the keywords "next generation probiotics" or "next generation probiotic", a search was conducted in PubMed on April 11, 2025. The search was restricted to English-language articles, yielding a total of 347 publications. Subsequently, a rigorous screening process was implemented: studies focusing on genetically engineered bacteria, investigations related to the respiratory tract or upper gastrointestinal tract, as well as review articles were excluded. Only those studies that were animal or human clinical trials targeting the gut microbiota were retained. Based on these criteria, a curated list of BCMs was established by integrating both the BCMs with Safe status and next-generation probiotic (NGP) candidates mentioned above (Table S9).

#### 3. Core microbiome

Core microbiome analysis was conducted based on de novo assembled high-quality MAG profiles (Wu et al., 2024). To construct the co-abundance network, we utilized prevalent genomes shared by more than 50% of the samples. The SparCC algorithm was employed to calculate correlations between the genomes, based on their abundances across donors, with 100 permutations performed to assess statistical significance (Friedman and Alm, 2012). Correlations with *P* < 0.01 were retained for further analysis. The original network was visualized using Cytoscape v3.7.1 (Shannon et al., 2003), followed by the implementation of the Analyze Network function to calculate node attributes. Nodes with both Betweenness Centrality = 0 and Closeness Centrality = 0 were filtered out to construct a core-network, with the constituent nodes that were defined as core microbiome. The layout of nodes and edges was determined using an edge-weighted spring embedded layout, with the correlation coefficient serving as the weight for each connection. Nodes in the network were clustered using weighted correlation network analysis (WGCNA), employing mean linkage hierarchical clustering conducted in R (Langfelder and Horvath, 2008).

### Functional profiling

Microorganisms harboring antibiotic resistance traits and VFs are regarded as potential threats to human health. Hence, we use the Resistance Gene Identifier (RGI v6.0.1) in command line mode (rgi bwt) to predict resistome(s) from all valid shotgun sequencing reads based on reference sequences in Comprehensive Antibiotic Resistance Database (CARD) and KMA algorithms (Alcock et al., 2023). Based on antimicrobial-resistant pathogens prioritized by the EU Council, the U.S. CDC, the China Antimicrobial Surveillance Network (CHINET), as well as those commonly monitored in FMT donor screening, ARGs related to 11 commonly monitored drug-resistant microorganism groups were investigated, including meticillin-resistant *Staphylococcus aureus* (MRSA), extended-spectrum beta-lactamase (ESBL)-producing Enterobacterales group, Carbapenem-resistant Enterobacterales (CRE) group, carbapenem-resistant *Acinetobacter* (CRA), *Candida auris* (*C. auris*), vancomycin-resistant *Enterococcus* (VRE), multidrug-resistant (MDR) *Pseudomonas aeruginosa* (PA), vancomycin-resistant *Staphylococcus aureus* (VRSA), carbapenem-resistant *Pseudomonas aeruginosa* (CRPA), multidrug-resistant coagulase-negative staphylococci (MRCNS), and multidrug-resistant *Acinetobacter baumannii* (MDR-AB) (Carlson, 2020).

VFs were classified by mapping the high-quality reads to the Virulence Factors of Pathogenic Bacteria Database (VFDB) (April 11, 2023) database using BWA (v0.7.17-r1198-dirty) (Jung and Han, 2022). SAMtools (v1.14) was subsequently utilized to calculate the covered loci and sequencing depth corresponding to the mapped genes in VFDB database.

Python scripts were specifically developed to quantify gene coverage, defined as the ratio of the total mapped region to the gene length. Resistome(s) and virulence factor genes with coverage greater than 70% was considered as positive results. Furthermore, the gene's abundance is determined based on its mean sequencing depth.

Protein-coding genes of HQMAGs were predicted using Prodigal v2.6.3. Functional annotation of HQMAGs was conducted by aligning against the Kyoto Encyclopedia of Genes and Genomes database (KEGG, v20230830) using Diamond (Version 0.8.35) with the BLASTP algorithm at an E-value threshold of 1e-5.

### Model construction

The classification model was constructed using Random Forest algorithm with Ntree set to 999. Subsequently, ten-fold cross-validation was implemented to verify node importance, and model performance was evaluated via receiver operating characteristic (ROC) curve analysis. The downloaded gut microbiome metagenomic data of healthy individuals from the HMP and CMP were used as the general healthy individuals (designated as NormalH). These data, along with those of the donor from China and the USA (designated as Donor), were used for model construction. The genomes of core microbiome of donors from China and the USA with redundancy removed (> 99% mean Nucleotide Identity, ANI) were employed as reference genomes to calculate the abundance of samples in the NormalH and Donor groups. Batch effects of core microbial communities across studies in China and the USA were corrected using the adjust_batch function in the MMUPHin package (Liu et al., 2022; Ma et al., 2022). This correction was based on relative abundance profiles of core microbiome with BioProject ID serving as the batch variable (Table S1). To preclude data leakage during cross-country external validation, batch effect correction was performed independently within each country. Specifically, batch effect correction was performed on the core microbiome profiles of datasets from China, including FMT donor cohorts and healthy individuals from the CMP. Analogously, the same procedure was applied to FMT donor cohorts from the USA and healthy participants derived from the HMP cohort.

### Quantification and statistical analysis

Statistical analysis was performed in the R (Version4.0.2) or Python (Version3.9.5) environment. The mean, standard deviation, and prevalence of microbiota or genes were calculated using pandas (Version1.4.2) and numpy (Version1.21.4). Principal coordinates analysis (PCoA) was conducted using the vegdist function (package vegan) for distance matrix computation, and the base cmdscale function for coordinate ordination. Analysis of Similarities (ANOSIM), a non-parametric method designed to evaluate inter-group similarities in high-dimensional datasets, was employed to identify differences in microbial communities between the two groups. Pairwise inter-group differences in alpha diversity were assessed via the Mann-Whitney U test. Fisher’s exact tests were implemented using R’s fisher.test function. Data points with absolute Z-scores > 3 were identified as outliers and removed prior to downstream analysis.

## Results

A total of 475 metagenome-sequenced fecal samples from healthy donors across five countries and 2,112 species identified by the metagenomic analysis were included in the present study (Tables S1 and S2).

### Genome-based safety assessment

#### 1. Characterization of disease-related microorganisms and virulence factors

Given the importance of pathogen screening for the safety of FMT administration, we assessed the distribution of 8 pathogenic genera and 65 pathogenic species in the “DRMs list” (Table S3). Three pathogenic bacterial species were detected in the gut microbiota of all donors, namely *Escherichia coli*, *Clostridium butyricum*, and *Streptococcus pneumoniae*. *E. coli* exhibited the widest distribution in donors from China, with a detection rate of 94.38%, followed by those from New Zealand (53.45%), the USA (43.81%), the Netherlands (35.44%), and Canada (28.21%). *C. butyricum* exhibited an entirely opposite distribution trend, being most prevalent in Canada (79.49%) and least prevalent in China (10.11%). Additionally, *E. coli* had the highest relative abundance in donors from China (0.3755%) while *C. butyricum* had the highest relative abundance in donors from Canada (0.0608%). In contrast, *S. pneumoniae* was detected in only one fecal sample from the USA with a relative abundance of 0.0022% (Fig. 1A).

To assess pathogenic potential, we aligned the high-quality reads against the VFDB database. None of the donors were found to harbor genes encoding Shiga toxin, Shiga-like toxins, or BoNTs according to ECDC criteria (Table S4) (De Rauw et al., 2019; Fabris et al., 2026), indicating the detected *E. coli* and *C. butyricum* lacked major exotoxin genes. Furthermore, we investigated the distribution of high-prevalence VFs (defined as > 50% prevalence in at least one country) in donor gut microbiota. The results demonstrated that these VFs were primarily associated with motility, capsule-mediated immune modulation, and adherence, whereas exotoxin-related genes were absent. These high-prevalence VFs were identified in the gut microbiota of donors from China, Canada, and the Netherlands, with contributions from bacterial species including *C. difficile*, *E. coli*, *Salmonella enterica*, *Shigella dysenteriae*, *S. sonnei*, *Streptococcus gordonii*, *S. sanguinis*, and *S. thermophilus* (Fig. 1B).

#### 2. Antibiotic resistance traits identification

Since drug-resistant microbes represent another critical factor affecting FMT safety, we investigate ARGs associated with 11 commonly monitored antimicrobial-resistant microorganism groups, among which we detected ARGs related to five clinically important resistant bacterial groups in donor fecal samples. The CRE-associated ARGs showed the highest prevalence, primarily carried by *E. coli* strains from China, with a prevalence range of 48.31–70.79%. In the other four countries, the prevalence range of these CRE-associated ARGs were 0–20.69%. The ARGs associated with VRE had the second-highest prevalence, mainly harbored by *Enterococcus faecalis* from Canada with a prevalence range of 0–58.97%, followed by New Zealand and the Netherlands with prevalence ranges of 0–50% and 0–39.24%, respectively. China and the USA showed relatively lower prevalence with both ranging from 0% to 20.22% (Fig. 1C, Table S5). Importantly, ARGs profile exhibited significant variations across countries (Fig. 1D, Table S6).

### Gut microbiota characteristics of donors from five countries

Species-level intestinal flora composition revealed that the gut microbial profiles of samples from China and the USA exhibited higher similarity based on hierarchical cluster analysis, whereas those from Canada, the Netherlands, and New Zealand clustered together with relatively consistent species compositions. *Faecalibacterium prausnitzii* was detected in over 95% of samples and showed the highest mean relative abundance (5.91%) among these highly prevalent taxa (Fig. 2A, Table S2). At phylum level, the gut microbiota of FMT donors from China and the USA are dominated by *Bacteroidetes*, whereas *Firmicutes* is the predominant phylum among FMT donors from Canada, the Netherlands, and New Zealand (Fig. S1).

No significant difference was observed in the Shannon index between samples from China and the USA, while Shannon index values of samples from each of the other three countries were significantly higher than those from both China and the USA (*P* < 0.05, Fig. 2B, Table S7).

Although the PCoA and ANOSIM results showed significant differences of microbial community structure among countries, the microbial communities of China and the USA (Group G1) were comparable (R = 0.1985, ANOSIM). On the other hand, the microbial communities of Canada, the Netherlands, and New Zealand (Group G2) exhibited similar characteristics (mean R = 0.1789, ANOSIM). Subsequent comparative analysis of similarities between the predefined G1 and G2 identified significant difference in microbial community structure between the two groups (*P* = 0.001, R = 0.2938, ANOSIM) (Fig. 2C, Table S8).

### Analysis of BCMs abundance and their distribution in donor fecal samples

Given the well-documented health benefits of BCMs, and the critical role of gut microbial diversity in gut homeostasis, we calculated both the species richness and total relative abundance of BCMs within the whole gut microbiota. A total of 151 species were included in the BCMs List compiled in this study (Table S9). The results showed that 56 species of BCMs were cumulatively detected across all samples (Fig. 3A). Interestingly, *F. prausnitzii*, exhibited the highest mean relative abundance among the detected probiotics, indicating the potential health benefits conferred by the gut microbiota of eligible donors (Fig. 3A and 3B).

Moreover, the analysis revealed 5–22 species of BCMs across all donor samples and no difference were observed between donors of China and the USA. Notably, gut microbiota of donors in both China and the USA showed significantly lower species richness of BCMs than that in Canada (9–19 species) and the Netherlands (8–22 species) (*P* < 0.01) but not New Zealand (6–17 species) (Fig. 3C, Table S10). The total relative abundance of BCMs, which ranged from 4.557% to 94.658% across all donors, was comparable between donors in China and the USA and significantly higher than that of other three countries (Fig. 3D, Table S10).

### Core microbiome analysis

Co-abundance network analysis was performed to explore the associations among high-prevalence (> 50%) HQMAGs and identify the core microbiome. The results demonstrated that two guilds (designated as cluster0 and cluster1) were generated for both China and the USA, whereas a single guild was formed for Canada, New Zealand, and the Netherlands (Fig. 4A). In both China and the USA, most inter-guild correlations were negative (329/373 and 1845/2774, respectively) while intra-guild correlations were predominantly positive (515/608 and 1486/1772, respectively). Taxonomic annotation of the core microbiome showed that nodes in cluster1 of both China and the USA were predominantly dominated by the *Lachnospiraceae* family, which was significantly higher than that in cluster0 (Fig. 4B, Table S11).

To explore the differences of functional profiles among core microbiome guilds, protein-coding genes were predicted for genomes within each guild and subsequently annotated against the KEGG database. PCoA was conducted based on the functional gene count profiles of each guild across KEGG level-3 pathways (Fig. 4C), indicating 3 separated clusters (cluster 0 from China and the USA, cluster 1 from China and the USA, and others). Notably, significant differences in functional gene composition were observed among these three groups (Fig. 4C). These findings further suggested similarities in the gut microbiota between donors from China and the USA. We further constructed an exploratory retrospective classifier based on features of the core microbiome to distinguish eligible FMT donors from general healthy individuals. After batch effect correction, the classifier constructed based on the features of core microbiome in Cluster1 demonstrated the best predictive performance during cross-country external validation (Table S12). In detail, when the dataset of China was used as the training set, the AUC values of the trained models for predicting the USA dataset were 0.764 (using core microbiome from cluster1). When the dataset of the USA served as the training set, the AUC values of the models for predicting the China dataset were 0.714 (Fig. S2A and S2B). In addition, the AUC values change to 0.77 and 0.713 when adding Shannon index into the model (Fig. S2C and S2D).

## Discussion

Our study analyzed gut metagenomic data from healthy FMT donors across 24 independent studies spanning 5 countries. Three pathogenic bacterial species, namely *E. coli*, *C. butyricum*, and *S. pneumoniae*, were detected in the donor intestines, but virulence annotation showed the absence of Shiga toxins, Shiga-like toxins, or BoNTs encoding genes in all samples. Additionally, BoNTs are primarily produced by *C. botulinum*, and rarely by other *Clostridium* species such as *C. argentinense*, *C. baratii*, and *C. butyricum* (Lonati et al., 2020; Rawson et al., 2023). These results indicate the limited pathogenicity of the detected *E. coli* and *C. butyricum* strains. *S. pneumoniae*, a common colonizer of the upper respiratory tract but not a typical resident of the intestinal microbiota, was detected in the fecal sample of one donor from the USA at a very low relative abundance of 0.0022%, which was far below the threshold for reliable confirmation of intestinal colonization (Engholm et al., 2017; Nishijima et al., 2025). Taken together with its ecological niche preference, this finding suggests that the detection of *S. pneumoniae* in this case is likely a false positive. Collectively, our findings demonstrate that the current donor screening methods are effective in preventing infections caused by the pathogenic organisms listed in the DRMs List.

However, it’s worth to note that critical limitations still exist in addressing ARG-related risks according to current donor evaluating process. The distribution of ARGs detected from fecal samples of FMT donors exhibited obvious geographic heterogeneity, which might be primarily attributed to distinct environmental resistome profiles across different countries (Osawa et al., 2026). Substantial evidence indicates that environmental ARG reservoirs can profoundly influence human-associated resistomes through multiple transmission pathways, such as dietary exposure and direct environmental contact, thereby shaping the observed geographical distribution of ARGs in the human gut (Berendonk et al., 2015; Wang et al., 2026; Xue et al., 2025). Rare but severe adverse events including bacteremia and death caused by transmission of extended-spectrum beta-lactamase (ESBL)-producing organisms from inadequately screened donors have been reported (DeFilipp et al., 2019), which enhanced the necessity of routine screening for drug-resistant pathogens in FMT donors. Indeed, existing screening methods based on bacteriological cultures or pathogen-specific PCR assays fail to completely eliminate ARGs associated with several high-priority drug-resistant bacteria (e.g., *TolC*, *marA*, *vanR* gene in *vanG* cluster), which might result in serious clinical consequences when the colonization occurs in immunocompromised recipients. This not only dampens the therapeutic effect of FMT but also poses a serious safety risk in clinical practice.

This study found that *F. prausnitzii* was the most widely distributed and the most abundant species among all donors, which is inconsistent with previous reports (Lee et al., 2024). This discrepancy may be attributed to the fact that this study focused on FMT donor data. As reported, *F. prausnitzii* exerts numerous probiotic functions in the human gut, such as producing short-chain fatty acids and helping the body resist inflammation, and thus is recognized as a next-generation probiotic (Cheng et al., 2024; Lee et al., 2020). These beneficial effects are consistent with the original intention of FMT donor screening.

Consistent with previous reports, we observed significant differences in gut microbiota characteristics across countries, indicating that gut microbiota was shaped by multiple environmental factors such as dietary structure, geographic environments, and other population-specific factors (Parizadeh and Arrieta, 2023). Interestingly, compared with donors from the other three countries, donors from China and the USA exhibited similarity across multiple dimensions, including phylum-level dominance of *Bacteroidetes*, alpha diversity, the species richness and total relative abundance of the detected BCMs, and the co-abundance patterns of core microbiome. These results support the biological plausibility of performing cross-population external validation of an exploratory retrospective classifier for FMT donors between China and the USA.

Furthermore, current donor screening strategies for FMT primarily rely on lifestyle questionnaire surveys, clinical assessments, and targeted detection of specific pathogens in stool and blood samples (Liu et al., 2025b; Ng et al., 2024). However, these conventional approaches lack standardized criteria based on the overall gut microbial composition, which might lead to significantly different safety and therapeutic outcomes of FMT. Building upon the development by Wu et al. (2024) of a genome-specific, database-independent, and interaction-focused core microbiome indicator for health and disease states, the current study made a preliminary attempt to explore the co-abundance patterns of the core microbiome among FMT donors across countries. Our results identified two distinct co-abundance patterns of core microbiomes in the gut of healthy donors. These patterns showed a distinct association with the dominant phylum of the gut microbiota. A *Firmicutes*-dominant community was characterized by one core microbiome guild. In *Bacteroidetes*-dominant communities, the core microbiome formed two guilds, suggesting divergent metabolic potentials (Turnbaugh et al., 2006).

Gut metagenomic data downloaded from the CMP and HMP, which served as the general healthy population, were combined with metagenomic data from donors in China and the USA for the exploratory retrospective classifier training and validation. Given the low donor eligibility rates (1.71–3.19% in China and ~3% in the USA), recruiting eligible donors from the general healthy population is challenging (He et al., 2021; Kassam et al., 2019; Zhang et al., 2022a). Therefore, employing CMP and HMP datasets to represent the general healthy population is reasonable. Additionally, to further improve the predictive performance of the exploratory retrospective classifier, we integrated the batch-effect-corrected features of core microbiome in cluster1 with the Shannon index during classifier reconstruction. However, these combined features yielded only negligible improvement in performance (delta AUC = 0.006 and -0.001), suggesting that cluster1 of core microbiome features are more representative than the Shannon index to be used to distinguish previously screened FMT donors from general healthy individuals. Despite that alpha-diversity was used as an important parameter for donor matching in previous study (Zhang et al., 2023), our results suggest that the co-abundance pattern of the core microbiome may provide an alternative perspective for distinguishing donors from general healthy individuals.

Collectively, our study underscores the potential value of including genome-based ARG profiling for donor exclusion. Although current donor screening strategies are effective in excluding DRMs harboring key virulence factors, they remain insufficient in addressing ARG-related risks, which deserves careful consideration in future protocol revisions. Metagenomic analysis of donor fecal samples enables comprehensive detection of both DRMs and ARG-harboring microorganisms, which effectively overcoming the inherent limitations of conventional screening approaches. Thus, our findings provide direct evidence that it is valuable that to incorporate metagenomic sequencing into FMT donor screening protocols.

As a retrospective descriptive study, this work has several limitations. First, due to data availability, only a relatively limited number of countries were included in the present analysis, which may introduce potential bias in certain conclusions. In addition, all analyses were performed using publicly available datasets retrieved from multiple independent studies. It’s inevitable that variations in geographic origin, sample collection procedures, DNA extraction protocols, sequencing platforms, sequencing depth, and original study design across different projects may introduce data heterogeneity and batch-related variations. Such discrepancies may shape microbial community profiles, co-abundance network patterns, ARGs detection outcomes, and cross-regional comparative results. Furthermore, the exploratory retrospective classifier lacks prospective clinical validation based on independent FMT donor cohorts. Future work should incorporate larger sample sizes and datasets from more diverse geographic regions so as to further explore the functional relevance of core microbiome clusters, as well as to validate the microbiota-based exploratory retrospective classifier in clinical trials of FMT. These follow-up efforts may provide incremental insights for standardizing FMT donor selection and help gradually optimize the safety profile of clinical practice of FMT.

## Figures and Tables

**Fig. 1.**
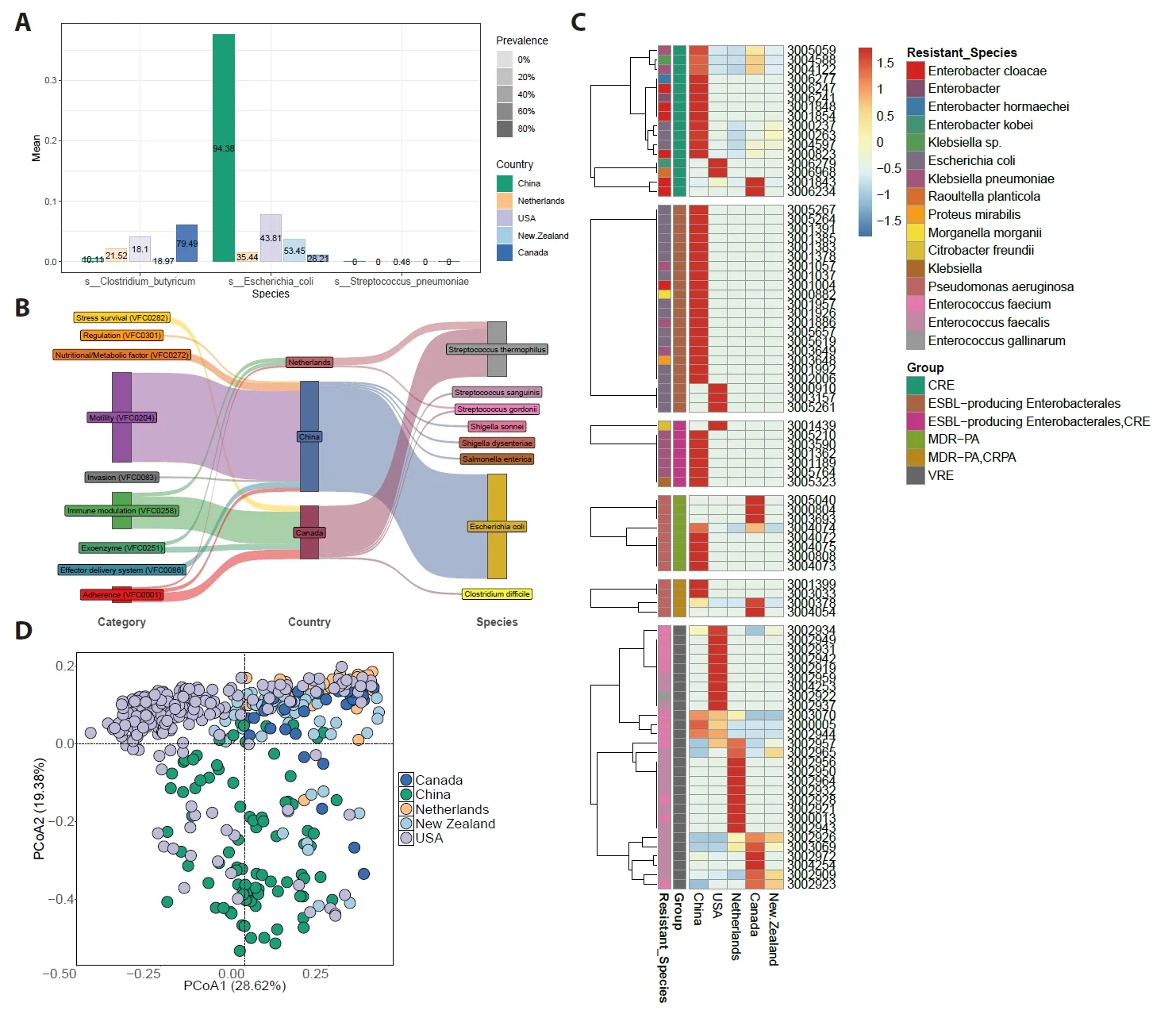
Characterization of DRMs, VFs, and ARGs in the gut microbiome of FMT donors. (A) Bar plot showing prevalence rates (numerical values within bars) and mean relative abundance (bar height) of DRMs. (B) Distribution of high-prevalence VFs. (C) Distribution of ARGs in five groups of detected monitored drug-resistant microorganisms. (D) PCoA for ARGs profile of donors across five countries.

**Fig. 2.**
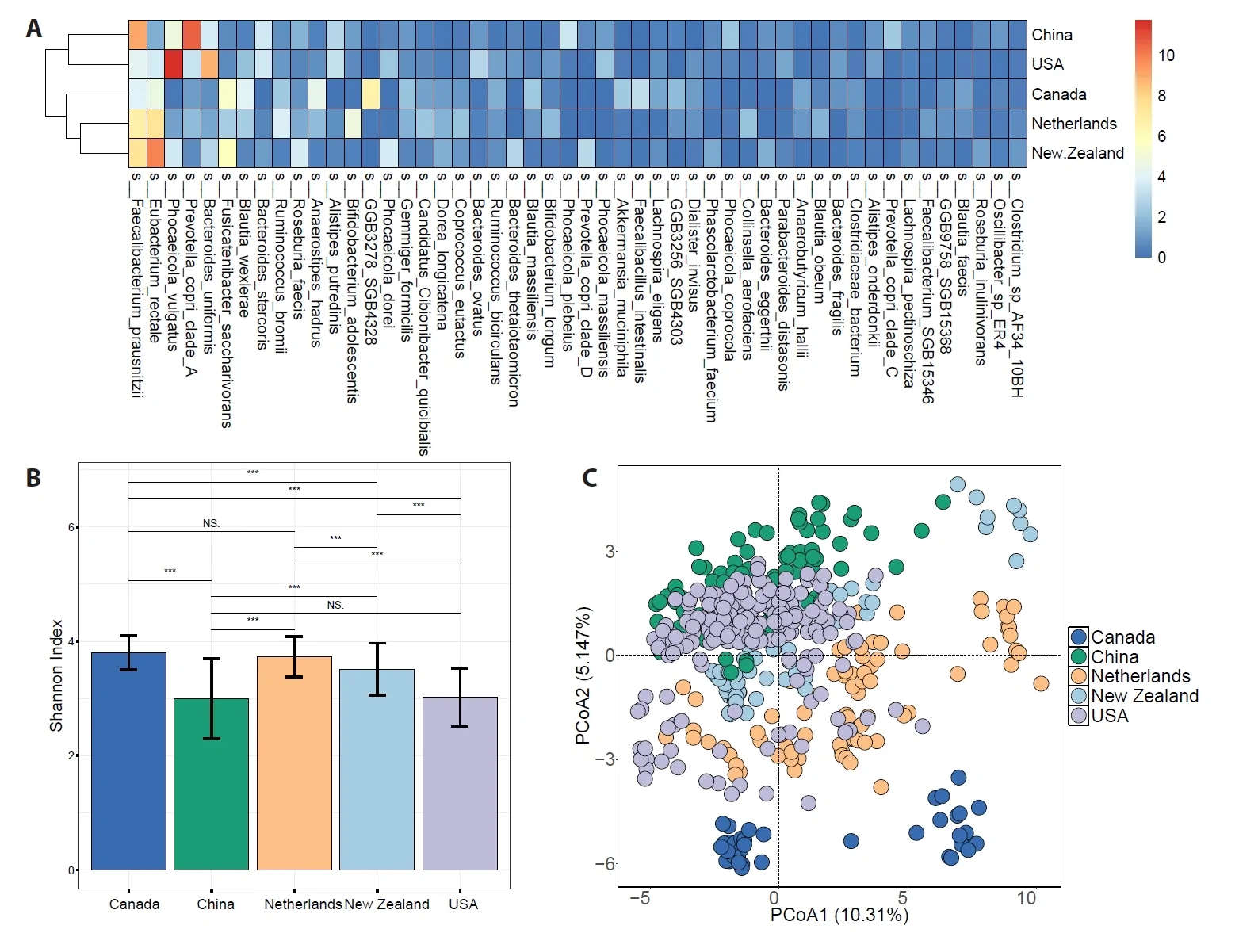
Gut microbiota composition and diversity of FMT donors. (A) Mean relative abundance of the top 50 bacterial species, ranked in descending order (left to right). (B) Pairwise Mann-Whitney U test for Shannon index between countries. (C) PCoA of fecal microbial communities across donors from five countries. NS *P* > 0.05, *P < 0.05, **P < 0.01, ***P < 0.001.

**Fig. 3.**
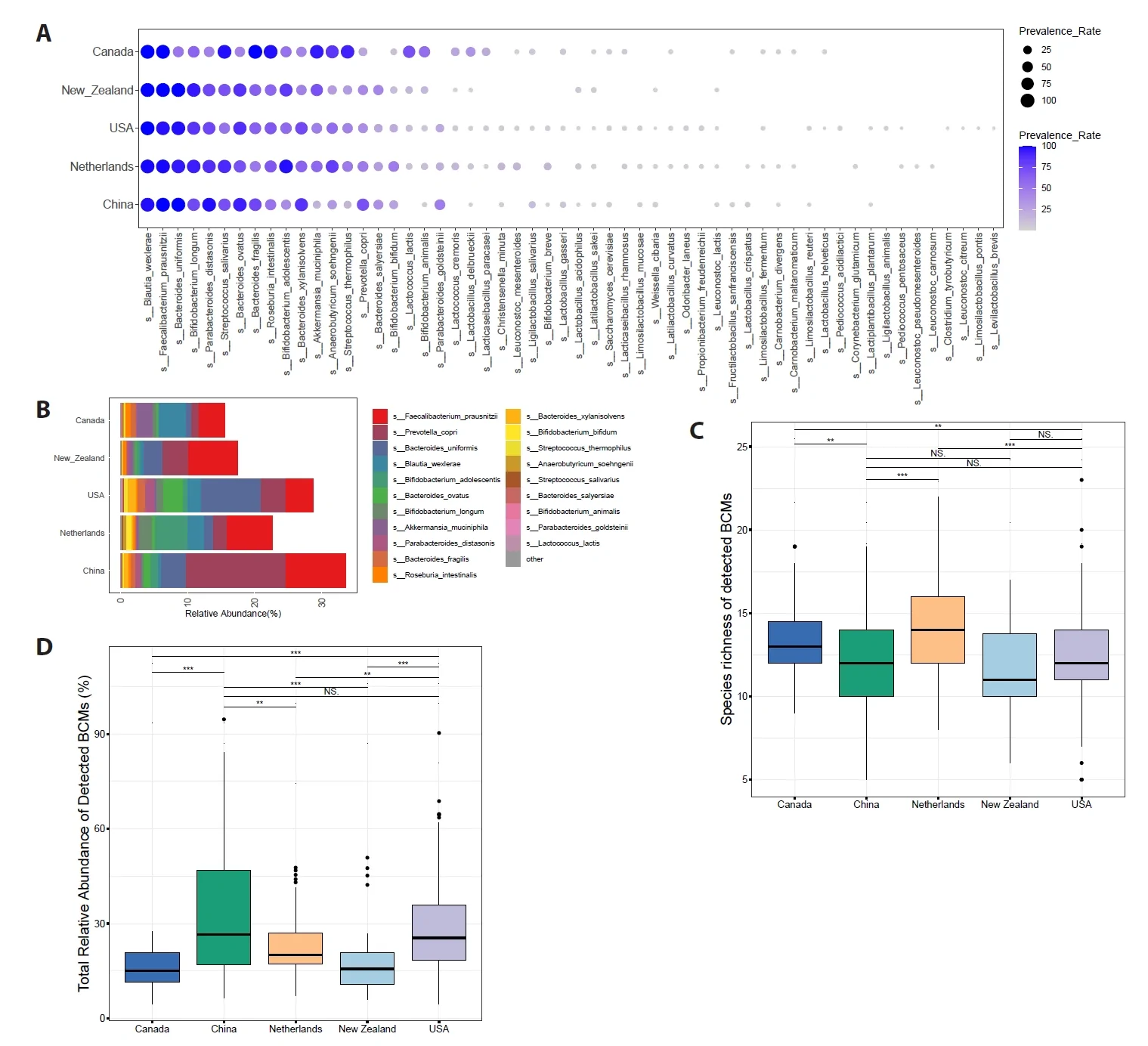
Distribution characteristics and abundance of detected BCMs in the gut microbiome of FMT donors. (A) The prevalence of detected BCM species across five countries. (B) The community composition bar plot illustrates the relative abundance of the top 20 taxa and remaining species grouped as "others". (C) Pairwise Mann-Whitney U test for species richness of detected BCMs between countries. (D) Pairwise Mann-Whitney U test for total relative abundance of detected BCMs between countries. NS *P* > 0.05, *P < 0.05, **P < 0.01, ***P < 0.001.

**Fig. 4.**
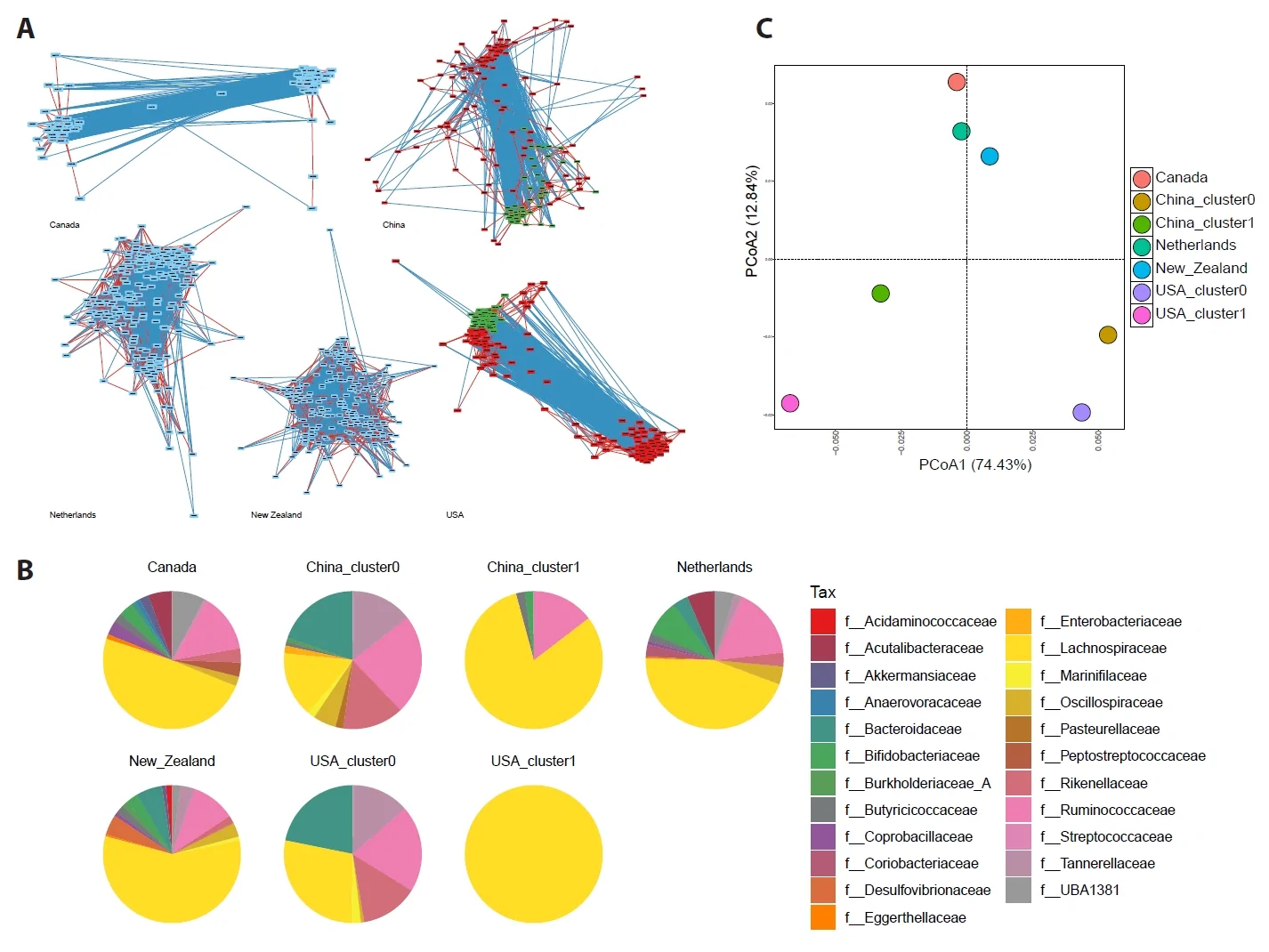
Core microbiome analysis of FMT donors. (A) Co-abundance networks based on HQMAGs of five countries. Networks were constructed using SparCC (retaining significant correlations, *P* < 0.01), with nodes represent HQMAGs (prevalence > 50%) colored by cluster membership (China/USA: red for cluster1, green for cluster0; Canada/New Zealand/Netherlands: blue for the single cluster). Edges represent correlations, with red and blue denoting positive and negative interactions, respectively. The network layout was determined using an edge-weighted spring-embedded algorithm, where correlation efficient served as the weight for positioning nodes and edges. (B) Taxonomic composition of core microbiome clusters at the family level. (C) PCoA of functional profiles in core microbiome clusters based on Bray-Curtis dissimilarity (KEGG pathway Level 3).

## Supplementary Information

Table S1. Basic information of samples from the 24 BioProjects included in this study

Table S2. Prevalence and mean relative abundance at species‑level

Table S3. Disease-related microorganisms list

Table S4. Prevalence of Virulence Factors (VFs)

Table S5. Prevalence of Antibiotic resistance traits

Table S6. Results of pairwise comparison analysis of similarities (ANOSIM) based on ARGs profile

Table S7. Shannon index of fecal microbiota from donors in 5 countries and their intra-country statistics, including original values, means, standard deviations, and Z-scores

Table S8. Results of pairwise comparison analysis of similarities (ANOSIM) based on species-level profile

Table S9. List of Beneficial commensal microorganisms

Table S10. Number of detected probiotic species and their total relative abundance from donors in 5 countries and their intra-country statistics, including original values, means, standard deviations, and Z-scores

Table S11. Fisher's Exact Test for the number of *Lachnospiraceae* family genomes among two clusters within China and USA, respectively

Table S12. Donor selection model based on core microbiome using random forest

Fig. S1. Phylum-level microbiota composition showing hierarchical clustering of taxa (left dendrogram) with bubble size representing prevalence and color intensity indicating mean relative abundance.

Fig. S2. Cross-countries external validation of Random Forest models (Ntree = 999) for gut microbiome-based predictions. (A) ROC curve of the model trained on dataset of China using core microbiome features from Cluster1 and validated on an independent the USA dataset. (B) Reciprocal validation with model trained on the USA dataset and tested ondataset of China. (C–D) Corresponding validations incorporating both Cluster1 features and Shannon diversity index.
