# Supplementary material for: A retrospective metagenomic analysis of fecal microbiota transplantation donors from five countries: Safety considerations for donor screening and core microbiome profiles of qualified donors
Source: J Microbiol. 2026 Sep 30;64(9):e2604010. doi: 10.71150/jm.2604010 (PMC13688126; doi:10.71150/jm.2604010)
Supplement: Supplementary file 1 [file jm-2604010-Supplementary-Fig-S1.pdf]

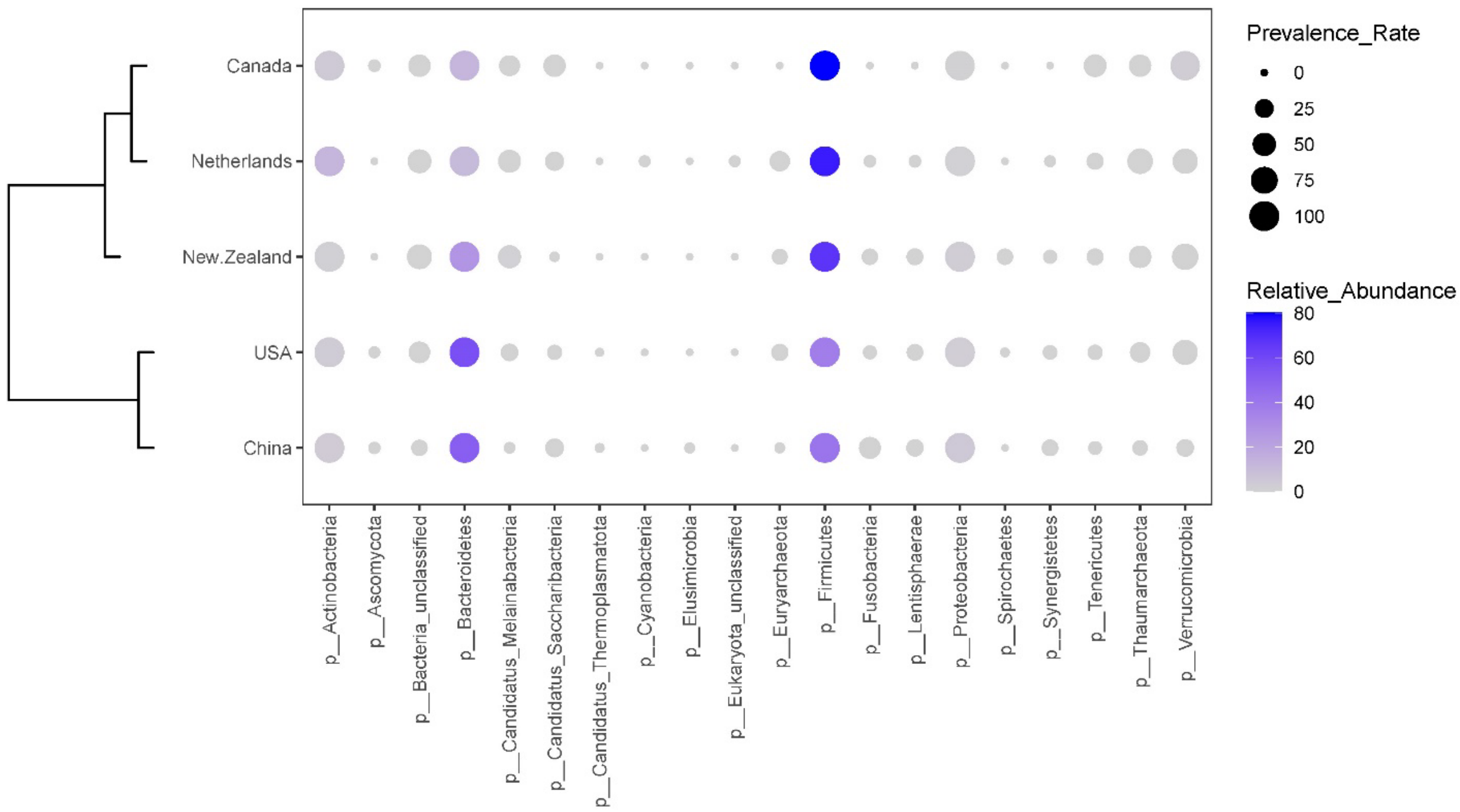

Fig. S1. Phylum-level microbiota composition showing hierarchical clustering of taxa (left dendrogram) with bubble size representing prevalence and color intensity indicating mean relative abundance.
