# Supplementary material for: A retrospective metagenomic analysis of fecal microbiota transplantation donors from five countries: Safety considerations for donor screening and core microbiome profiles of qualified donors
Source: J Microbiol. 2026 Sep 30;64(9):e2604010. doi: 10.71150/jm.2604010 (PMC13688126; doi:10.71150/jm.2604010)
Supplement: Supplementary file 2 [file jm-2604010-Supplementary-Fig-S2.pdf]

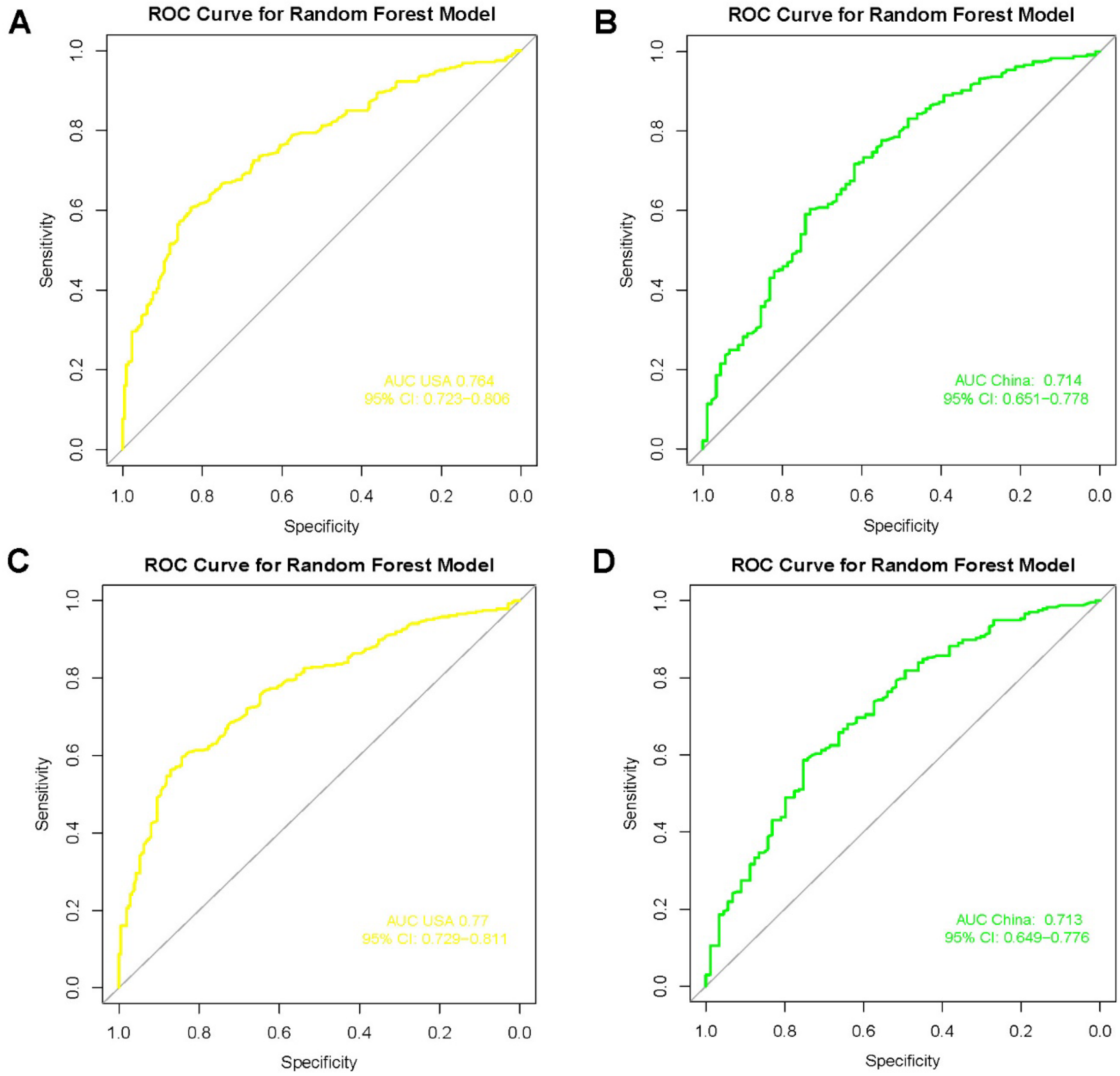

Fig. S2. Cross-countries external validation of Random Forest models (Ntree = 999) for gut microbiome-based predictions. (A) ROC curve of the model trained on dataset of China using core microbiome features from Cluster1 and validated on an independent the USA dataset. (B) Reciprocal validation with model trained on the USA dataset and tested ondataset of China. (C–D) Corresponding validations incorporating both Cluster1 features and Shannon diversity index.
